# Supplementary material for: Frequency and Prognostic Significance of Intertumoural Heterogeneity in Multifocal Jejunoileal Neuroendocrine Tumours
Source: Cancers (Basel). 2022 Aug 17;14(16):3963. doi: 10.3390/cancers14163963 (PMC9406343; doi:10.3390/cancers14163963)
Supplement: Supplementary file 1 [file cancers-14-03963-s001.zip › cancers-1853052-supplementary.pdf]

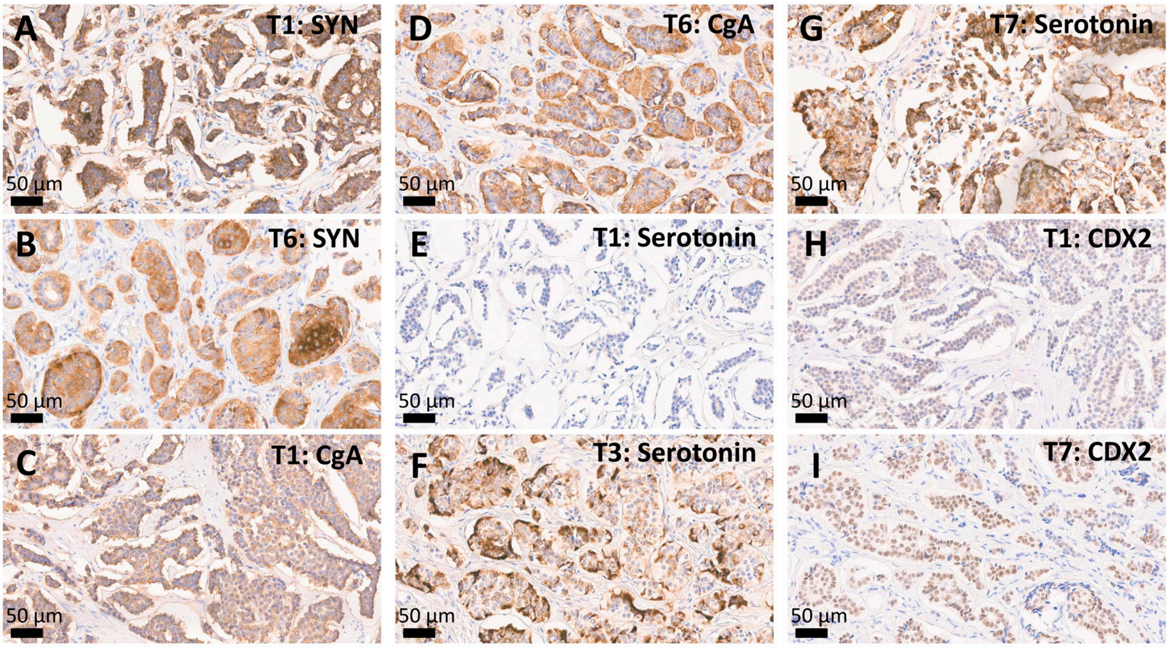

Figure S1: Expression of standard diagnostic markers in multifocal SI-NETs.

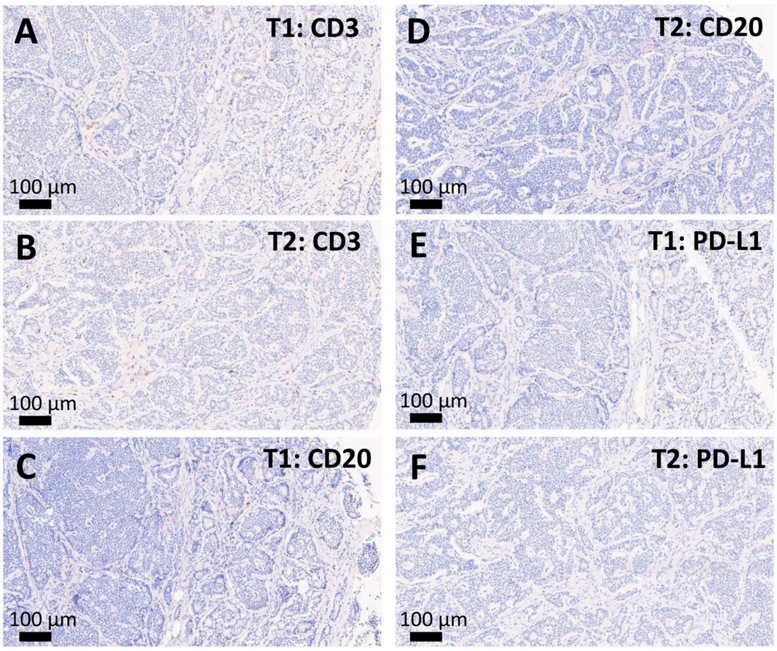

Figure S2: Contexture of the immunogenic microenvironment in multifocal SI-NET.

Supplementary Table S1: Clinicopathological characteristics of our cohort of multifocal SI-NET.

Gender	Age	tumour localization	Numer of primary tumours	pT (leading primary)	pN	pM	UICC-stage	R-status
male	56	Ileum	3	3	1/2	1	IV	0
male	63	Ileum	36	3	1/2	1	IV	1
male	62	Ileum	8	2	1/2	0	III	1
male	64	Ileum	11	3	1/2	1	IV	0
female	51	Ileum	12	3	1/2	1	IV	1
female	74	Ileum	3	3	1/2	1	IV	1
male	64	Ileum	2	1	1/2	1	IV	1
female	79	Ileum	13	3	1/2	0	III	1
male	67	Ileum	6	3	1/2	1	IV	0
female	63	Ileum	3	3	1/2	1	IV	1
male	48	Ileum	9	3	1/2	1	IV	1
female	71	Ileum	2	3	1/2	1	IV	1
male	72	Ileum	6	3	1/2	1	IV	1
female	47	Ileum	13	3	1/2	1	IV	1
male	62	Ileum	10	3	1/2	1	IV	0
female	59	Ileum	15	3	1/2	1	IV	1
female	61	Ileum	7	2	1/2	1	IV	1
male	41	Ileum	12	3	1/2	0	III	1
female	73	Ileum	2	2	1/2	0	IV	1
male	41	Jejunum	2	3	0	0	II	1
female	78	Ileum	13	3	1/2	1	IV	1
male	76	Ileum	2	3	0	1	IV	0
female	46	Ileum	10	4	1/2	1	IV	1
male	62	Ileum	3	3	1/2	1	IV	1
female	63	Ileum	2	4	1/2	1	IV	0
male	27	Ileum	2	3	1/2	1	IV	1
female	63	Ileum	3	3	1/2	1	IV	1
male	68	Ileum	2	3	1/2	1	IV	1

Supplementary Table S2: Clinicopathological characteristics of our control cohort of unifocal SI-NET.

Sex	Age	localization	WHO-grade	pT	pN	pM	UICC stage
w	55	Ileum	G1	T3	N1	M1	4
m	36	Ileum	G1	T3	N1	M1	4
m	50	Ileum	G1	T3	N1	M1	4
m	69	Ileum	G1	T3	N1	M1	4
m	28	Ileum	G2	T3	N1	M1	4
m	60	Ileum	G1	T3	N1	M1	4
m	57	Ileum	G1	T2	N1	M1	4
m	53	Ileum	G1	T3	N1	M1	4
m	49	Ileum	G1	TX	N1	M1	4
m	49	Ileum	G1	T4	N1	M1	4
w	67	Ileum	G1	T3	N0	M1	4
m	57	Ileum	G1	T3	N1	M1	4
m	43	Ileum	G1	T3	N1	M1	4
w	66	Ileum	G1	T3	N1	M1	4
m	92	Ileum	G1	T3	N1	M1	4
w	44	Ileum	G1	T2	N1	M1	4
m	40	Ileum	G1	T3	N1	M1	4
m	76	Ileum	G1	T3	N1	M1	4
w	56	Ileum	G1	T3	N0	M1	4
w	66	Ileum	G1	T3	N1	M1	4
m	64	Ileum	G2	T2	N1	M1	4
w	60	Ileum	G1	T4	N1	M1	4
m	68	Ileum	G1	T3	N1	M1	4
w	71	Ileum	G1	T2	N1	M1	4
w	61	Ileum	G1	T2	N1	M1	4
m	63	Ileum	G1	T3	N1	M1	4
m	80	Ileum	G1	T3	N1	M1	4
m	64	Ileum	G1	T3	N1	M1	4
w	57	Ileum	G1	T3	N1	M1	4
w	68	Ileum	G1	T3	N1	M1	4
w	50	Ileum	G1	T3	N1	M1	4
m	60	Ileum	G1	T3	N1	M1	4
m	58	Ileum	G1	T3	N1	M1	4
m	59	Ileum	G1	T3	N1	M1	4
m	82	Ileum	G1	T4	N1	M1	4
w	53	Ileum	G1	T4	N1	M1	4
w	58	Ileum	G1	T4	N1	M1	4
m	53	Ileum	G1	T3	N1	M1	4
w	73	Ileum	G3	T0	N0	M1	4
m	46	Ileum	G2	T3	N1	M1	4
w	65	Ileum	G1	T3	N1	M1	4
m	49	Ileum	G1	T4	N1	M1	4
w	66	Ileum	G2	T3	N1	M1	4
m	45	Ileum	G1	T3	N1	M1	4
m	49	Ileum	G1	T3	N1	M1	4
m	64	Ileum	G1	T3	N1	M1	4
w	77	Ileum	G2	T3	N1	M1	4
w	44	Ileum	G1	T3	N1	M1	4
w	70	Ileum	G1	T3	N1	M1	4
w	63	Ileum	G1	T3	N0	M1	4
m	73	Ileum	G3	T3	N1	M1	4
m	43	Ileum	G2	T3	N1	M1	4
w	59	Ileum	G1	TX	N1	M1	4
w	61	Jejunum	G2	T3	N1	M1	4
w	75	Ileum	G1	T2	N1	M1	4
m	65	Ileum	G1	T3	N1	M1	4
w	68	Ileum	G2	T3	N1	M1	4
m	60	Ileum	G1	T4	N1	M1	4
m	60	Ileum	G1	T4	N1	M1	4
m	67	Ileum	G1	T4	N1	M1	4
w	56	Ileum	G1	T3	N0	M1	4
m	77	Ileum	G2	T3	N1	M1	4
m	56	Ileum	G1	T1	N1	M1	4
w	51	Ileum	G1	T2	N2	M1	4
m	64	Ileum	G1	T3	N1	M1	4
m	49	Ileum	G1	T4	N1	M1	4
m	52	Ileum	G1	T3	N1	M1	4
w	66	Ileum	G2	T2	N1	M1	4
m	50	Ileum	G2	T3	N1	M1	4
w	64	Ileum	G1	T3	N1	M1	4
m	49	Ileum	G1	T3	N2	M1	4
w	45	Ileum	G2	T3	N1	M1	4
m	51	Ileum	G1	T3	N1	M1	4
m	60	Ileum	G1	T3	N1	M1	4
m	64	Ileum	G2	TX	N1	M1	4
m	63	Ileum	G3	TX	N1	M1	4
w	41	Ileum	G1	T4	N1	M1	4
w	54	Ileum	G1	T4	N0	M1	4
m	61	Ileum	G1	T3	N1	M1	4
w	64	Ileum	G1	T3	N1	M1	4
m	77	Ileum	G2	T3	N1	M1	4
m	63	Ileum	G1	T2	N1	M1	4
m	73	Ileum	G1	T3	N1	M1	4
m	88	Ileum	G2	T4	N1	M1	4
w	68	Ileum	G1	T3	N1	M1	4
w	54	Ileum	G1	T4	N1	M1	4
m	54	Ileum	G2	T3	N1	M1	4
w	61	Ileum	G1	T3	N1	M1	4
